# The cost burden of Crohn’s disease and ulcerative colitis depending on biologic treatment status – a Danish register-based study

**DOI:** 10.1186/s12913-021-06816-3

**Published:** 2021-08-18

**Authors:** Sarah Alulis, Kasper Vadstrup, Jens Olsen, Tine Rikke Jørgensen, Niels Qvist, Pia Munkholm, Andras Borsi

**Affiliations:** 1 Janssen-Cilag, Bregnerødvej 133, 3460 Birkerød, Denmark; 2 Incentive, Holte, Denmark; 3 LEO Pharma, Ballerup, Denmark; 4 Surgical Department A and IBD Care, Odense University Hospital, Odense, Denmark; 5 Gastroenterology Department, North Zealand University Hospital, Frederikssund, Denmark; 6 Janssen-Cilag, High Wycombe, UK

**Keywords:** Epidemiology, National register data, Health economy, Societal burden, IBD, CD, UC

## Abstract

**Background:**

Patients diagnosed with inflammatory bowel disease may be treated with biologics, depending on several medical and non-medical factors. This study investigated healthcare costs and production values of patients treated with biologics.

**Methods:**

This national register study included patients diagnosed with Crohn’s disease (CD) and ulcerative colitis (UC) between 2003 and 2015, identified in the Danish National Patient Register (DNPR). Average annual healthcare costs and production values were compared for patients receiving biologic treatment or not, and for patients initiating biologic treatment within a year after diagnosis or at a later stage. Cost estimates and production values were based on charges, fees and average gross wages.

**Results:**

Twenty-six point one percent CD patients and ten point seven percent of UC patients were treated with biologics at some point in the study period. Of these, 46.4 and 45.5 % of patients initiated biologic treatment within the first year after diagnosis. CD and UC patients treated with biologics had higher average annual healthcare costs after diagnosis compared to patients not treated with biologics. CD patients receiving biologics early had lower production values both ten years before and eight years after treatment initiation, compared to patients receiving treatment later. UC patients receiving biologics early had lower average annual production values the first year after treatment initiation compared to UC patients receiving treatment later.

**Conclusions:**

CD and UC patients receiving biologic treatment had higher average annual healthcare costs and lower average annual production values, compared to patients not receiving biologic treatment. The main healthcare costs drivers were outpatient visit costs and admission costs.

## Background

Inflammatory bowel diseases (IBD), Crohn’s disease (CD) and ulcerative colitis (UC), are chronic diseases that affect people of all ages; but the majority of newly diagnosed patients are adolescents and those in early adulthood [1]. The cause of IBD remains unknown, however elements such as environment, genetics, and immunoregulatory factors are all thought to be associated [2]. Disease severity is classified as being mild, moderate or severe and the treatment patients may receive depends on this. Typically, patients with moderate to severe disease will receive biological treatment, depending on several medical and non-medical factors. As each patient’s IBD presents differently, treatment needs to be tailored to their specific situation [2].

Due to the chronic nature of IBD and its symptoms, quality of life is greatly impacted. An online survey across 25 national IBD associations found that 56 % of patients with CD felt that their disease affected their career and 17 % believed that CD caused their relationship to end [3]. In addition to the personal and emotional effects of IBD, a patient’s ability to work is often impacted. Studies show that having UC is associated with higher indirect costs related to loss of productivity [4], including the areas of sick leave, shorter working days, and early retirement. A previous study conducted in the United Kingdom found that the majority of UC patients experienced professional challenges related to their choice in work and the amount of time they could spend working [5]. Therefore, the aim of the present study was to explore the average annual healthcare costs and production values of patients ten years before their IBD diagnosis and in the eight-year period after initiating biologic treatment in Denmark.

## Methods

### Study population and study design

This retrospective population-based study explored the costs of having an IBD diagnosis, stratified on CD, UC, and biologic treatment status. Data on all Danish residents was retrieved from the Danish Civil Registration System (CRS) [6], that includes all citizens and residents with a civil personal registration number, enabling an identity-secure linkage of information between the national registries. Patient specific data was collected from the National Health Service Register (NHSR) [6–8], the Danish National Patient Registry (NPR) [7, 9, 10], the Cause of Death Register [7] and the Danish Longitudinal Database on Employment (DREAM) [11].

The DREAM database, owned by the Danish Ministry of Employment, includes information on weekly labour market transfer payments for all Danish citizens, since 1991 [11]. Only individuals receiving a labour market related social benefit payment are included in the database in the corresponding year. Hence, individuals that were employed, full-time the entire year are not included in the database. Yearly employment rates were estimated using the DREAM database.

The study population included all adults above 18 years of age diagnosed with CD or UC between 2003 and 2015 with the following selection criteria: (1) Individuals with at least two hospital contacts (admissions, outpatient or emergency room visits) collected from the NPR, with a primary or secondary diagnosis of CD or UC using the International Classification of Diseases 10th edition (ICD-10) code K50 and K51 and with at least one of the registrations defined as the primary diagnosis; (2) patient with no hospital contacts related to CD or UC during 1994–2002 (wash-out period). Further, index date was defined as the first hospital contact including either admission, outpatient or emergency room visit with diagnosis of CD or UC. Patients diagnosed with UC followed by a CD diagnosis were considered as diagnosed with CD. The study design, other analyses and results have previously been published [12–14].

The patients diagnosed with CD and UC were categorized into two groups: (1) Those who received CD or UC related biologic treatment with at least one hospital contact with a registered biologics treatment code in the period 2003–2016, and (2) those who did not receive CD or UC related biologic treatment in the period 2003–2016. Four biologic treatments were available during the study period: infliximab, adalimumab, vedolizumab and golimumab and were identified in the NPR using their treatment code. Those receiving biologic treatment were further divided into two sub-groups: 1a) Patients that initiated biologic treatment within the first year from their CD or UC diagnosis, and 1b) patients that initiated biologic treatment more than a year after diagnosis.

Patients were censored (excluded) at death and at end of follow-up (2016). In the year of death or the end of follow-up, the individual was included with a weight corresponding to the fraction of the year data were available for them.

### Costs and production value

Healthcare costs and production values were extracted from the NHSR, the NPR and the DREAM database. All contacts with the primary healthcare and hospital sector are collected in the NHSR and the NPR, respectively. The primary healthcare sector includes general practitioners, private practicing medical specialists and other private practicing healthcare professionals such as chiropractors and psychologists. The hospital sector includes admissions, outpatient and emergency room visits. The gross fee paid for each contact with a healthcare professional came from the NHSR, and outpatient (DAGS) and Diagnosis Related Group (DRG) charges for each contact were extracted from the NPR. Total healthcare costs included primary sector contacts, outpatient contacts, hospital admissions and gross fees (primary care sector), and charges (outpatient contacts and admissions) were applied as unit cost estimates.

In Denmark, the prescription of and treatment with biologics occurs solely within the hospital sector (i.e., 100 % publicly financed hospital drugs). IBD patients are not treated in private hospitals, as private insurance does not cover the funding of biologics. Treatment with intravenous therapies, such as infliximab and vedolizumab, always takes place in a hospital, usually during an outpatient visit. Treatment with subcutaneous treatments, such as adalimumab and golimumab, may be given in the hospital or the patient may administer it at home. If administrated at home, the patient needs to pick up the medicine at least every three months from the hospital, in which it will be registered as an outpatient contact. The drug costs are either included in the cost of the admission or in the outpatient contacts (i.e. the DRG/DAGS charge). All biologics included in this analysis are included in the national treatment guidelines, therefore implying that compassionate use programs are not relevant and/or do not exist for this patient population.

The average annual production value was estimated using weekly employment data from the DREAM database on employment. Using this database, the annual employment rate for all patients included was estimated as the percentage of the year employed and unemployed, respectively. Production values were then estimated by multiplying the annual employment rate with a gender specific gross average annual wage, adjusted for the number of effective weekly working hours [15, 16]. For estimation of production value, only individuals between the age of 18 and 65 each year were included, as they are considered to constitute the work force.

Fees in the NHSR and DAGS and DRG charges in the NPR were inflated using the relevant combined price and wage index for healthcare services, estimated by the Danish Regions [17]. All costs and production values are presented in the 2016 price level. All costs are reported in Euros with the exchange rate of €1 = DKK 7.5.

### Statistical analyses

Two main analyses were conducted. The first analysis investigated the average annual costs and production value of CD and UC patients, before and after diagnosis. Patients receiving biologic treatment after diagnosis were compared to patients that did not receive biologic treatment after diagnosis. The second analysis consisted of the average annual costs and production value in the period after biologic treatment initiation for the sub-groups of CD and UC patients on biologic treatment. Patients that initiated biologic treatment within the first year after diagnosis (i.e. early treatment initiation) were compared to those patients that initiated biologic treatment more than a year after diagnosis (i.e. late treatment initiation).

For the first analysis, average annual costs and production value per individual were calculated in the ten-year period prior to the CD and UC incidence date, and the eight-year period after the incidence date. As the two patient groups may not be comparable in terms of age and gender distribution, a linear regression model was conducted and adjusted for age and gender. For the second analysis, average annual costs and production value per individual were calculated in the year prior to the date of biologic treatment initiation and then up to eight years after the biologic treatment initiation date. This was done for the two sub-groups of patients receiving biologic treatment within the first year of diagnosis, and for patients receiving biologic treatment more than one year after diagnosis.

All statistical analyses were conducted in SAS version 9.4 (SAS Institute Inc, Cary, NC, USA) on Statistics Denmark’s research computers via a remote server.

## Results

Between 2003 and 2015, a total of 9,019 CD and 20,913 UC patients were identified in the NPR. Of these, 2,351 CD (26.1 %) and 2,248 UC patients (10.7 %) received diagnosis related biologic treatment at some point during the study period, Table 1. Among the CD diagnosed patients who were treated with biologics, 1,091 (46.4 %) initiated treatment within the first year after diagnosis, whereas 1,260 (53.6 %) initiated treatment more than a year after diagnosis. For the UC patients treated with biologics, 1,022 (45.5 %) initiated treatment within the first year after diagnosis and 1,226 (54.5 %) patients began treatment later.

**Table 1:** Number of persons initiating biologic treatment and population size by year, CD and UC patients

Year -	2003	2004	2005	2006	2007	2008	2009	2010	2011	2012	2013	2014	2015	2016	Total^a^
CD patients initiating biologic treatment	0	5	20	67	166	168	201	257	236	252	243	274	280	182	2,351
Incident CD population, cumulated	695	1,418	2,140	2,809	3,493	4,197	4,876	5,670	6,472	7,213	7,878	8,534	9,019		9,019
% of incident CD population initiating biologics	0.0 %	0.4 %	0.9 %	2.4 %	4.8 %	4.0 %	4.1 %	4.5 %	3.6 %	3.5 %	3.1 %	3.2 %	3.1 %		26.1 %
UC patients initiating biologic treatment	0	1	4	36	99	109	164	192	246	248	275	343	322	209	2,248
Incident UC population, cumulated	1,610	3,286	4,938	6,513	8,063	9,736	11,604	13,432	15,399	17,011	18,503	19,858	20,913		20,913
% of incident UC population initiating biologics	0.0 %	0.0 %	0.1 %	0.6 %	1.2 %	1.1 %	1.4 %	1.4 %	1.6 %	1.5 %	1.5 %	1.7 %	1.5 %		10.7 %

The population of CD/UC patients initiating biologic treatment each year include all patients derived from the incident population receiving treatment for the first time the year initiated biologic treatment

^a^Totals include the total number of patients in each population in the entire period 2003–2015

### Treatment of biologics vs. not treated with biologics

**Fig. 1:**
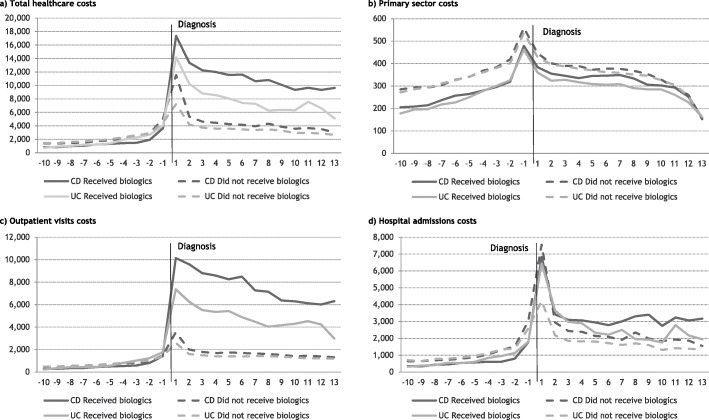
Average annual individual healthcare costs before and after diagnosis, stratified on biologic treatment status. **a** Total healthcare costs, **b** Primary sector costs, **c** Outpatient visits costs, **d** Hospital admissions costs

Overall, CD and UC patients who received biologic treatment had higher average annual healthcare costs after diagnosis compared to patients who did not receive biologic treatment, Fig. 1. The first and second year after diagnosis, the average total healthcare costs among CD patients receiving biologic treatment were €5,828 and €8,013 higher compared to patients not receiving biologic treatment. Among UC patients receiving biologic treatment, the average total costs in the first and second year after diagnosis exceeded those of the UC patients not receiving biologic treatment with €6,948 and €6,066 respectively. Eight years after diagnosis, the differences decreased to €6,618 for CD and €2,412 for UC patients.

The main healthcare cost drivers were outpatient visits and admission costs, which were overall higher among patients receiving biologics treatment, Fig. 1. Admission costs were higher in the first four years after diagnosis for UC patients who were treated with biologics compared to patients not treated with biologics. Among CD patients, admission costs were markedly higher the third, fourth and fifth year after diagnosis. The years prior to diagnosis, CD and UC patients not treated with biologics tended to have slightly higher admission costs.

**Fig. 2:**
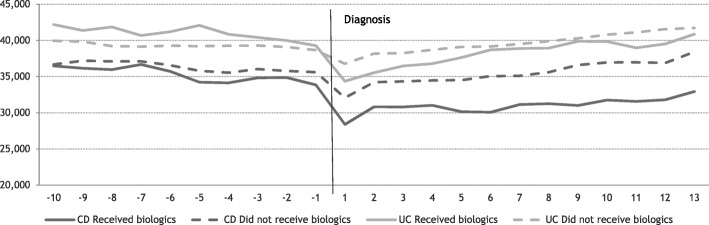
Average annual individual production value before and after diagnosis, stratified on biologic treatment status

Regarding primary sector costs, these were similar for the two patient groups. However, during the entire study period, patients that did not receive biologic treatment incurred higher costs compared to the patients that did, Fig. 1. The average annual production value per individual showed patients receiving biologic treatment had lower production value after their diagnosis, compared to patients that did not receive biologic treatment, Fig. 2.

The regression analyses adjusted for age and gender illustrated that after diagnosis, costs from outpatient visits and admissions were significantly lower among patients not receiving biologic treatment compared to patients receiving biologic treatment, Table 2. Patients not receiving treatment with biologics had a significantly higher production value after diagnosis compared to patients on biologic treatment. The primary sector costs, adjusted for age and gender, were higher some years before diagnosis, compared to patients treated with biologics, Table 2. There was however no statistically significant difference in primary sector costs between the patient groups after disease diagnosis.

**Table 2:** Adjusted differences (receiving biologics or not) of individual annual healthcare costs and production value (Euros, 2016 prices)

Years from diagnosis	Outpatient visits		Hospital admissions		Primary sector		Production value	
	CD	UC	CD	UC	CD	UC	CD	UC
-10	111	145*	96	249	12	33*	345	-1380
-9	64	71	118	88	23	28*	740	-1142
-8	22	9	49	142	21	37*	214	-1989*
-7	32	43	26	-14	8	31*	-390	-655
-6	17	34	69	89	15	33*	0	-890
-5	15	28	23	42	14	24*	488	-1426
-4	72	-150*	86	2	26	16	473	-149
-3	71	-278*	285*	17	28*	13	538	48
-2	-53	-395*	193	-170	39*	9	547	76
-1	-164	-619*	457*	70	29*	13	1419*	393
1	-6711*	-4914*	-367	-2908*	6	-7	3611*	3460*
2	-7698*	-4859*	-1133*	-1962*	-7	3	3451*	3810*
3	-7127*	-4203*	-1259*	-1618*	-4	-8	3609*	2887*
4	-7060*	-4089*	-1293*	-1507*	11	-2	3713*	3077*
5	-6662*	-4171*	-1289*	-1041*	-16	2	4592*	2802*
6	-6883*	-3637*	-1167*	-962*	-8	-1	5184*	1831*
7	-5751*	-3171*	-1462*	-1249*	-9	-4	4483*	2322*
8	-5689*	-2768*	-1440*	-621*	3	8	5127*	2858*
9	-4981*	-2959*	-1651*	-717*	14	13	6618*	2358*
10	-4976*	-3077*	-1169*	-695*	-6	-2	6491*	2835*
11	-4806*	-3347*	-1807*	-1629*	-28	4	6412*	3735*
12	-4721*	-3086*	-1730*	-1161*	-20	-2	6366*	3443
13	-5183*	-1848*	-2015*	-832	-30	-28	7050*	2425

*p ≤ 0.05

Overall, the difference in healthcare costs the first and second year after diagnosis, when only considering the statistically significant results, were €6,711 and €15,542 for CD patients and €7,822 and €6,821 for UC patients. This is higher than what was shown in the unadjusted comparison.

### Timing of biologic treatment

**Fig. 3:**
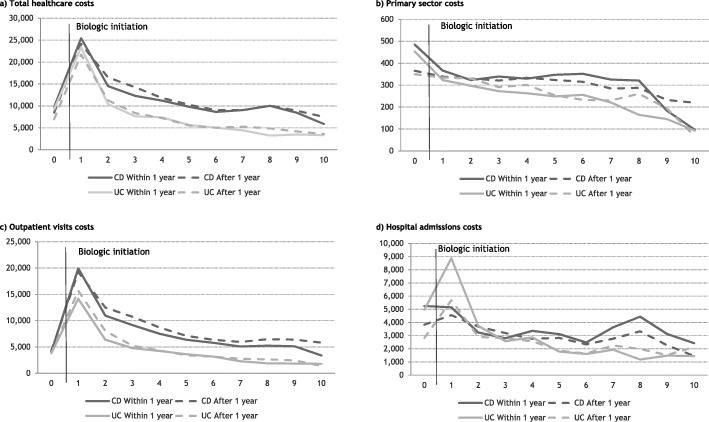
Average annual individual healthcare costs after biologic initiation, stratified on timing of treatment initiation related to diagnosis. **a** Total healthcare costs, **b** Primary sector costs, **c** Outpatient visits costs, **d** Hospital admissions costs

In general, the healthcare costs associated with early and late biologic treatment initiation were not very different. CD patients initiating biologic treatment earlier had on average €1,309 higher total healthcare costs the first year after treatment initiation, compared with CD patients initiating biologic treatment later, Fig. 3.

UC patients who initiated biologic treatment later had €1,692 lower total average healthcare costs (age and gender adjusted) during the first year after treatment initiation, compared to those with early initiation, Table 3. There were no significant differences in healthcare costs in the first year after treatment initiation among CD patients when adjusting for age and gender differences.

**Table 3:** Adjusted difference, individual annual healthcare costs and production value (patients initiating biologics within and after 1 year) (Euros, 2016 prices)

Years from biologic initiation	Outpatient visits		Hospital admissions		Primary sector		Production value	
	CD	UC	CD	UC	CD	UC	CD	UC
0	232	-75	-1462*	-2200*	-122*	-114*	1938	-169
1	-656	1497*	-661	-3189*	-30	-1	2963*	3459*
2	1563*	1583*	421	-872*	6	18	1314	1174
3	1592*	434	385	237	-19	7	2314	547
4	1193*	69	-596	-324	3	27	2998*	810
5	744	-253	-281	94	-22	0	3941*	441
6	569	-7	-133	22	-34	-29	5141*	-256
7	893	404	-894	429	-39	-3	2605	-129
8	1204	635	-1121	887	-31	88*	4313	-1880
9	1244	429	-795	-133	48	33	3329	172
10	2430*	-505	-1221*	649	135*	-34	4626	7436

^*^p≤0.05

Overall, patients initiating biologics early had higher primary sector costs and admission costs before treatment initiation compared to patients initiating biologics later. Outpatient visit costs differed significantly among CD patients in the second, third and fourth year after treatment initiation. UC patients initiating treatment later had significantly lower admission costs in the first and second year after treatment initiation compared to patients initiating treatment early.

**Fig. 4:**
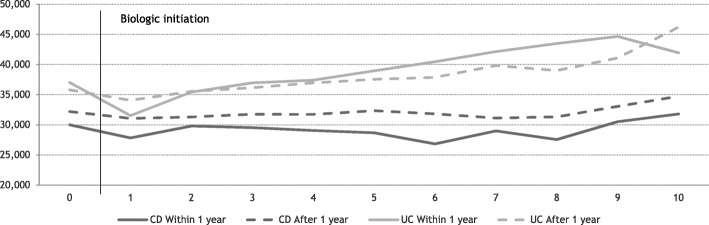
Average annual individual production value after biologic initiation, stratified on timing of treatment initiation related to diagnosis

The average annual production values among CD patients treated with biologics within the first year, were lower both before and after biologic treatment initiation compared to patients who received biologic treatment later, Fig. 4. UC patients who received biologic treatment within the first year after diagnosis had a lower average annual production value the first year after treatment initiation compared to patients that received biologic treatment more than a year after diagnosis. Adjusting for age and gender, the difference in production value for UC patients was significant the first year after treatment initiation with an estimated higher production value of €3,459 compared to patients initiating treatment later. CD patients initiating treatment after one year had production values exceeding €2,963 - €5,141 in the first, fourth, fifth and sixth year after treatment initiation compared to earlier treated patients, Table 3.

## Discussion

The general level and development of the average annual healthcare costs and production value of CD and UC patients before and after their diagnosis has previously been described in this population [12, 13]. Therefore, the current study compared the differences in healthcare costs and production value of CD and UC patients depending on if they received biologic treatment or not. More than a quarter of the CD study population received biologic treatment at some point in the period 2003–2016 after their diagnosis and more than 10 % received biologic treatment after a UC diagnosis.

This study showed that CD and UC patients that received biologic treatment had higher average annual healthcare costs and lower average annual production values compared to patients that did not receive biologic treatment. Despite biologics being used more and more [14], this study implies that patients treated with a biologic still constitute a more severely affected patient population compared to those patients that did not initiate biologic treatment. However, registry data does not include disease severity information for IBD-patients, therefore this study was not able to control for it. Our findings that receiving treatment with biologics leads to higher overall healthcare costs in IBD patients are supported by other German and Canadian real world evidence studies [18, 19].

This study also zoomed in on the differences in costs and production value between patients initiating biologic treatment within the first year after diagnosis, and patients initiating biologic treatment more than one year after diagnosis. The differences observed between receiving early and late treatment appeared marginal and non-systematic. However, after adjusting for age and gender, some of the results were significant and suggested that patients treated later had higher outpatient costs and production value, and lower admission costs the first years after treatment initiation, compared with earlier treated patients.

We need to also acknowledge that a subgroup of our cohort may have initiated biologic treatment very late after receiving an IBD diagnosis. Therefore, these patients may have a different disease course or severity and we cannot exclude that their healthcare cost may be lower, due to a shorter treatment period with more efficacious treatments. This study used a washout period of 10 years prior to inclusion, and we cannot exclude, that some patients diagnosed prior to the wash-out period might have been included. The number of patients that may have been included is considered to be very low due to clinical practice for IBD-patients in Denmark which includes a close follow-up, irrespective of disease activity. In a previous study using the same cohort, we have shown that biologic treatment does not change the cumulative surgical rate significantly in IBD-patients, and this may to some extent explain the increased costs in patients treated with biologics [20].

A previous study on the costs of CD and UC revealed higher healthcare costs after diagnosis compared to an age and gender matched control group free of IBD. Healthcare costs were especially high in the first years after diagnosis [12]. This trend is most likely explained by the difference in healthcare costs in the first year of patients initiating biologic treatment early and patients initiating treatment later (i.e. patients initiating biologic treatment later are beyond the “expensive” first year).

This study demonstrated that costs remained higher for patients treated with biologics more than a decade after diagnosis and that annual production values of patients receiving biologics were also lower a decade after diagnosis.

An important strength of this study is that selection bias and information bias is limited as the registries include all Danish residents, all data is prospectively collected, and the data quality is generally considered to be high. In addition, it is possible to follow patients for a potentially long period. Another strength is the broad perspective taken which includes both costs of outpatient visits and admissions, and primary healthcare sector costs and production values.

A limitation of the present study could be misclassification as we have had to rely on the accuracy of the ICD-10 coding in the NPR to identify CD and UC patients. In general, the reliability and validity of the diagnosis registration in the NPR are assessed to be adequate [9, 21, 22]. However, to our knowledge there are no studies explicitly validating the registration of CD and UC diagnosis codes. Furthermore, the first biologic treatment for CD and UC was infliximab, which was introduced to the European market in 1999, followed by adalimumab in 2003. Our study period was 2003–2016 covering the majority of the period with available biologic treatments, however more patients have initiated biologic treatment in the more recent years. Another potential limitation to this study is that it did not capture quality of life measures, which are important factors when assessing patients’ healthcare resource utilization, production values, and in determining the success or failure of a biologic treatment.

Finally, the end of follow up for each case was either due to death or the end of the data period, which was 2016. This study was not able to include information on emigrations meaning cases that emigrated from Denmark at some point during the study period will not have any registered costs after emigration, thus reducing the average cost estimate. Consequently, average costs might have been underestimated.

## Conclusions

CD and UC patients receiving biologic treatment had higher average annual healthcare costs and lower average annual production values, compared to patients not receiving biologic treatment. For the healthcare costs, the main cost drivers were outpatient visits and admissions. The timing of biologic treatment (early after diagnosis vs. later) does influence the costs, but the cost differences are minor.

## Data Availability

The data that support the findings of this study are available from Statistics Denmark’s Research Service, but restrictions apply to the availability of these data, which were used under license/authorisation for the current study, and so are not publicly available. Additional data analyses are however available from the authors upon reasonable request and with permission of Statistics Denmark’s Research Service.

## Abbreviations

IBD: Inflammatory bowel diseases
CD: Crohn’s disease
UC: Ulcerative colitis
CRS: Danish Civil Registration System
NHSR: National Health Service Register
NPR: National Patient Register
DREAM: Danish Longitudinal Database on Employment
ICD-10: International Classification of Diseases 10th edition
DAGS: Danish outpatient
DRG: Diagnosis Related Group
